# A Photothermally Enhanced Vancomycin-Coated Liquid Metal Antimicrobial Agent with Targeting Capability

**DOI:** 10.3390/bioengineering10070748

**Published:** 2023-06-22

**Authors:** Bo Wang, Sen Chen, Xuyang Sun, Xiaohui Shan, Xiyu Zhu, Bo Yuan, Hongzhang Wang, Gang Zhou, Jing Liu

**Affiliations:** 1 School of Biological Science and Medical Engineering, Beihang University, Beijing 100083, China; wangbo_2019@buaa.edu.cn (B.W.);; 2 Department of Biomedical Engineering, School of Medicine, Tsinghua University, Beijing 100084, China; 3 Center of Double Helix, Tsinghua Shenzhen International Graduate School, Tsinghua University, Shenzhen 518055, China; 4 Beijing Key Lab of Cryo-Biomedical Engineering, Technical Institute of Physics and Chemistry Chinese Academy of Sciences, Beijing 100190, China

**Keywords:** liquid metal, vancomycin, targeted antibacterial, photothermal conversion

## Abstract

The targeted antimicrobial efficacy of Vancomycin decreases significantly over time due to bacterial resistance, whereas Ga-based liquid metals, which are less prone to inducing bacterial resistance, face challenges in achieving targeted antimicrobial effects. To tackle these issues, a highly efficient antimicrobial agent with targeting properties has been developed by combining Ga-based liquid metals and Vancomycin. Moreover, the performance of this antimicrobial agent can be greatly enhanced through the use of near-infrared light. Microscopic observations reveal that Vancomycin can be effectively encapsulated on the surface of liquid metal, facilitated by the presence of the oxide layer. The resulting core–shell structured antimicrobial agent demonstrates notable targeted antimicrobial effects against *S. aureus*. Antibacterial tests indicate that Vancomycin effectively improves the antibacterial properties of pure liquid metal. Additionally, this study unveils the excellent photothermal conversion capabilities of liquid metal, enabling the antimicrobial agent exposed to 808nm near-infrared light to exhibit significantly strengthened bactericidal performance. In this scenario, the antimicrobial agent can achieve nearly 100% effectiveness. This work enriches the investigation of integrating Ga-based antimicrobial agents with traditional antibiotics, showcasing promising antibacterial effects and establishing the groundwork for subsequent clinical applications.

## 1. Introduction

Antibiotics play a vital role in maintaining human health, with vancomycin (Van) being a representative glycopeptide antibiotic known for its strong interaction with a wide range of Gram-positive bacteria [1]. On the premise of ensuring safety, Van has significant therapeutic effects on severe infections, including lung infections and skin and soft tissue infections, which are caused by *Staphylococcus aureus* (*S. aureus*), methicillin-resistant *S. aureus* (MRSA), and other bacteria [2,3,4]. The mechanism of action involves inhibiting bacterial growth and reproduction [5]. More specifically, Van can inhibit the generation of phospholipids and peptides and interfere with the synthesis of bacterial cell walls, thus achieving strong antibacterial effects against specific bacteria [6,7]. Considering that Van is a narrow-spectrum antibiotic, it has targeted antibacterial properties [8,9,10,11]. It interacts with bacterial cells through hydrogen bonds between the heptapeptide backbone of vancomycin and the d-alanyl-d-alanine dipeptide on the cell wall [12]. However, the phenomenon of antibiotic abuse has become increasingly serious in recent years [5,13,14], and bacteria that are resistant to Van have emerged [15,16,17,18], making it challenging to achieve the desired bactericidal effect with a single antibiotic in many cases. Therefore, it is crucial to maintain or improve the long-term antibacterial effectiveness of Van.

Gallium-based liquid metal (LM) has gained significant attention across various fields due to its exceptional biocompatibility [19] and its dual properties as a metal and a fluid [20]. Its applications range from thermal management [21] and flexible electronics [22,23] to injectable electronics [24], soft robots [25], and biomedicine [26]. Notably, its unique properties have provided solutions to many problems in the field of biomedicine, such as tumor treatment [27,28], drug delivery [29,30], vascular imaging [31], and biosensing [32,33]. In addition, previous studies have also demonstrated that LM displays antibacterial properties [34,35,36]. The mechanism lies in the highly similar electronegativities, ionic radii, ligand affinities, and electron and coordination geometries between Ga^3+^ and Fe^3+^. This similarity enables Ga^3+^ to replace Fe^3+^ and thus interfere with Fe^3+^-related physiological activities of biological systems, resulting in antibacterial effects [37,38,39]. More importantly, Ga-based antimicrobials are less prone to bacterial resistance. Ga^3+^ does not participate in redox reactions under physiological conditions, avoiding interference with oxygen transport in red blood cells and minimizing toxicity. This property allows for safe in vivo usage [40]. Despite these advantages, achieving targeted antimicrobial properties with Ga-based LM remains challenging due to the passive diffusion of Ga^3+^, which limits its localization at the desired site.

To address the aforementioned issues, we have developed a nanomaterial system that combines the antibacterial properties of LM and Van, resulting in a superior antibacterial effect with targeted characteristics. Furthermore, the antibacterial performance of this material system can be enhanced through the photothermal effect of LM [41,42,43,44,45]. Specifically, we achieved the coating of vancomycin onto the surface of LM using ultrasound-assisted methods, taking advantage of the presence of the oxide film. The unmodified liquid metal nanoparticles exhibited instability and deformity under light stimulation, but their stability significantly improved after modification with Van. Extensive testing has confirmed the excellent photothermal conversion efficiency of LM under near-infrared (NIR) light stimulation. Microscopic observations demonstrated the targeted effect of this antibacterial material system on bacteria. In practical applications, the antibacterial effect of Van-coated LM was more effective compared to single LM. Furthermore, the use of NIR light greatly enhanced the antibacterial effect of this material system, even achieving 100% antibacterial efficacy. This fundamental antimicrobial study lays the foundation for subsequent clinical application and holds promise for addressing bacterial resistance and improving therapeutic outcomes.

## 2. Materials and Methods

### 2.1. Materials

LM was composed of 75.5% Ga and 24.5% In by weight. A fixed ratio of Ga and In was placed in a beaker, heated until completely melted into liquid state, and then cooled to room temperature for later use. Van and other chemical drugs were purchased from Macklin (Shanghai, China) and used without purification.

### 2.2. Synthesis of LM and LM@Van

To prepare the LM@Van nanoparticles, 100 mg LM, 50 mg vancomycin, and 10 mL ddH_2_O were mixed in a 20 mL glass bottle. The above solution was sonicated in an ice bath for 30 min (5 s work, 5 s interval). Afterward, free Van was removed via centrifugation at 8000 rpm for 5 min, and the precipitates were redispersed in water. LM nanoparticles were synthesized according to the above steps without adding vancomycin.

### 2.3. Characterization of LM and LM@Van

The morphology of the nanoparticles was characterized using a transmission electron microscope (TEM, Tecnai Spirit, FEI, Hillsboro, OR, USA) and scanning electron microscope (SEM, SU-8010, Hitachi, Tokyo, Japan). The hydrodynamic diameters and zeta potential of the LM nanoparticles were measured using Mastersizer 3000 (Malvern, UK).

### 2.4. Photothermal Effect of LM and LM@Van

LM and LM@Van solution (0.2 mL) in 96-well plates were irradiated with 808 nm laser for 5 min, respectively. The infrared thermal images were captured using an IR camera.

### 2.5. Bacterial Culture and Antibacterial Evaluation In Vitro

The Gram-positive bacterium *Staphylococcus aureus* (*S. aureus*) was grown overnight at 37 °C, washed three times with PBS, and adjusted to OD_600_ to 1. Subsequently, 200 μL *S. aureus* suspension was added into a 48-well plate containing LM or LM@Van with a final concentration of 0.1 mg/mL (PBS as control). The mixed suspension was incubated in the incubator (37 °C, 120 min) with or without NIR laser (1 W/cm^2^) stimulation for 5 min. Bacteria samples were diluted step by step and a 100 μL dilution was spread onto the LB agar solid medium. The plates were cultured in the 37 °C incubator for 20 h, and the growth of the colonies was observed.

### 2.6. Live/Dead Staining

The bacterial viability was imaged using the live/dead staining method. Bacteria samples were incubated with different nanoparticles of 0.2 mL at 37 °C for two hours. The NIR-treated groups were stimulated using an 808 nm laser (1 W/cm^2^) for 5 min. Then, *S. aureus* cells were incubated with 1 μM live/dead dye at room temperature for 15 min, and observed using a laser scanning confocal microscope (FV3000, Olympus, Tokyo, Japan).

### 2.7. Bacterial Morphological Characterization

*S. aureus* was incubated with different concentrations of nanoparticles for two hours and the bacteria were collected via centrifugation at 10,000 rpm for 2 min and then washed with PBS three times. Afterward, bacterial samples were dehydrated for 10 min with sequentially concentrated ethanol solutions, and then observed via TEM and SEM.

### 2.8. Cytotoxicity Testing In Vitro

The cytotoxicity of LM@Van against NIH 3T3 cells was studied using the cell counting kit-8 (CCK-8) assay. NIH 3T3 cells were placed in 96-well plates with 100 μL of medium containing 5000 cells and cultured overnight at 37 °C with 5% CO_2_ using complete medium (10% FBS and 1% P/S). Different concentrations of LM@Van were added and incubated for 12 h or 24 h. Finally, cells were washed with PBS and incubated in a cell culture chamber at 37 °C for 2 h after adding 10 μL CCK-8 per well. Cell viability was measured using a microplate reader with absorbance at 450 nm.

## 3. Results and Discussion

**Figure 1:**
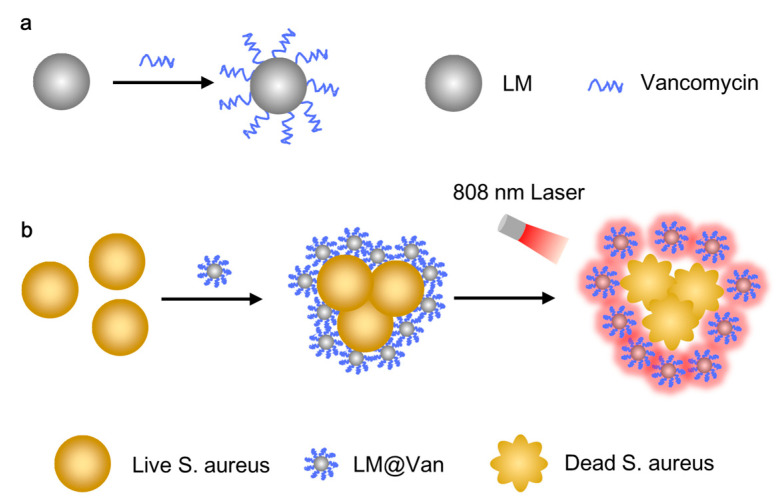
Diagram for antibacterial material’s structural composition and its antibacterial principle. (**a**) Schematic diagram of the LM@Van droplets. (**b**) Schematic diagram of targeted antibacterial and photothermal enhancement in LM@Van droplets.

The dispersion of LM into nanodroplets offers advantages in terms of increasing its contact area with bacteria, thereby achieving better bactericidal effects. In this study, ultrasound was used to disperse LM. The structural composition of the antimicrobial nanomaterial system is depicted in Figure 1a, illustrating that the nanomaterial system consists of a core made of liquid metal and a shell made of Van. The targeted antibacterial ability and photothermally enhanced antibacterial properties of this nanomaterial system are shown in Figure 1b. Van on the surface of LM has the function of targeting *S. aureus*, resulting in the enrichment of antibacterial nanoparticles on the surface of *S. aureus* and achieving more efficient bactericidal effects. Furthermore, under 808 nm NIR light stimulation, the highly efficient photothermal conversion performance of LM nanoparticles leads to the deformation and death of the bacteria. Consequently, the antibacterial performance of this nanomaterial can be significantly improved.

**Figure 2:**
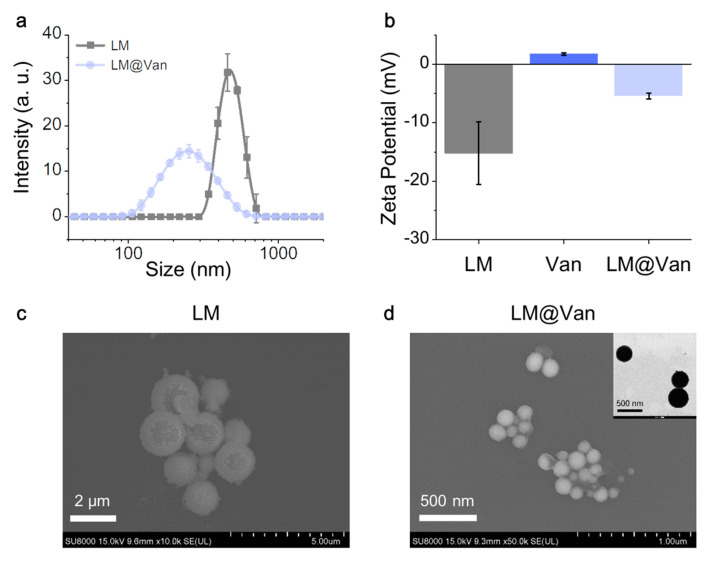
Characterization of particle size and morphology of antibacterial materials. (**a**) Particle size distribution of LM droplets before and after being coated by Van; (**b**) zeta potential analysis of LM, Van, and LM@Van; (**c**) microscopic surface morphology of pure LM; (**d**) the microscopic surface morphology of LM@Van. Inserted in the upper right corner is the transmission electron microscope image of LM@Van.

The particle size of antibacterial nanomaterials plays a crucial role in their application. To assess the particle size distribution of LM droplets and LM@Van droplets, dynamic light scattering (DLS) testing was performed. The results are shown in Figure 2a, which indicates that the average particle size of LM nanoparticles without surface modification is 500 nm, while the particle size of LM nanoparticles modified with Van is about 250 nm. Such a result shows that the particle size of LM droplets after modification is significantly reduced, indicating that the addition of Van is beneficial to the dispersion of LM droplets. In order to explore the underlying mechanism, the surface electrical properties of LM and Van were tested. Considering that zeta potential is a measure of mutual repulsion or attraction between nanoparticles, the zeta potentials of LM, Van, and LM@Van particle solutions were tested separately. The experimental results are shown in Figure 2b, demonstrating that the zeta potential of unmodified LM nanoparticles is −15 mV, while the potential of Van is +3 mV due to the presence of amino groups. Particles with opposite zeta potentials will attract each other, thereby achieving the encapsulation of Van on the surface of LM particles.

Further test results showed that the zeta potential of LM nanoparticles modified with Van shifted significantly toward the positive direction, approximately—5 mV, partially confirming the above analysis. The reason for the difference in zeta potential between the two is that Van has lots of amino groups on its surface, while LM nanoparticles have an oxide layer on their surface. Figure 2c,d indicate the apparent morphology of the antibacterial nanoparticles, indicating that the modified nanoparticles are smaller than the unmodified nanoparticles. Additionally, SEM experiments showed that a film covered the surface of the LM nanoparticles, which was Van surface modification. In order to observe the Van shell layer of approximately 10 nm thickness more intuitively, a transmission electron microscope (TEM) was used and the experimental results are shown in the upper right corner of Figure 2d and Figure S2.

We conducted additional tests on the nanoparticles using UV-visible absorption and IR absorption spectroscopy. Figure S1a illustrates the UV-visible absorption of Van and LM@Van. Van exhibits a distinct absorption peak near 280 nm, which originates from the phenyl groups in Van. LM@Van has absorption at all wavelengths, with a clear absorption peak at 300 nm and a red shift compared to Van, indicating that Van was successfully attached to the LM surface and the agglomeration phenomenon occurred. IR studies were carried out to reveal the different functional groups which are responsible for the interaction of Van on the LM surface. Figure S1b shows the IR spectra of Van and LM@Van. LM@Van has distinctive characteristic peaks on Van, such as O-H stretching vibration (3400 cm^−1^), C=O vibrational peak (1643 cm^−1^), and C-O vibrational peak (1241 cm^−1^). In addition to this, LM@Van shows strong peaks at 945 cm^−1^ and 636 cm^−1^ due to the stretching vibration of the -C-NH-amino group, while H atoms and O atoms in the LM surface oxide layer form hydrogen bonds. Hence, the addition of peaks indicates the interaction of vancomycin on the surface of LM.

The biocompatibility of nanoparticles is a crucial factor that should not be overlooked, as various factors including size distribution, morphology, surface charge, surface chemistry, and capping agents can influence their biological activity [46,47]. It is known that Ga has low toxicity, but only a few cases have been reported [48]. Numerous experimental results have shown that Ga with a large size is weakly toxic in the non-ionic state. The cytotoxicity test results of LM@Van at different concentrations showed that the nanoparticles had good biological safety, maintaining over 83% cell viability (Figure S1c). Therefore, LM@Van has excellent biocompatibility.

**Figure 3:**
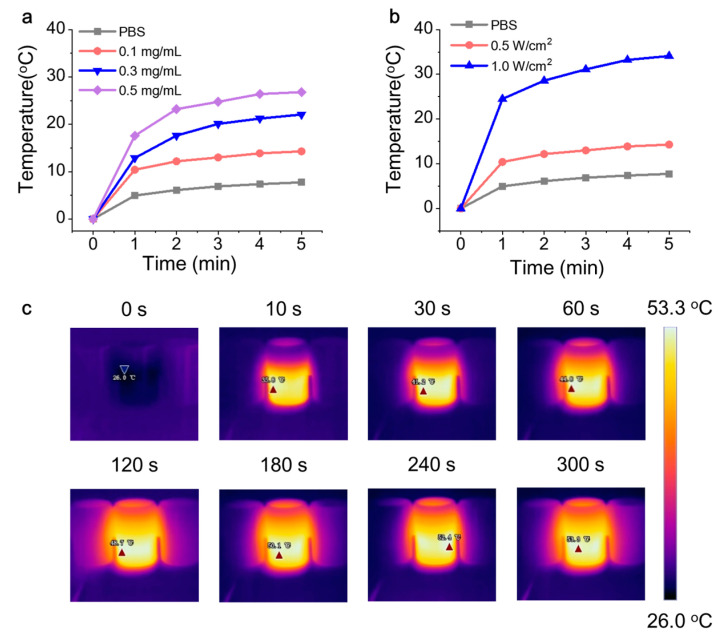
Photothermal conversion of LM@Van nanoparticle solution. (**a**) Temperature variation curves of nanoparticles with different concentrations at the same optical power density (0.5 W/cm^2^); (**b**) temperature variation curve for different optical power densities at fixed concentration (0.1 mg/mL); (**c**) NIR imaging of LM@Van nanoparticle solution under 808 nm laser irradiation.

The photothermal effect is crucial for enhancing the antibacterial performance of LM nanodroplet solutions. LM@Van nanoparticles have better photothermal efficiency, and this material is very stable and can be stimulated cyclically. Thus, we characterized the photothermal performance of this antibacterial material. Figure 3a shows the temperature curves of LM@Van nanoparticle solutions at different concentrations under the same light power density. As the light exposure time increased, the temperature rapidly increased and then reached a stable trend. Furthermore, as the concentration of the LM@Van nanoparticle solution increased, the temperature change gradually increased, reaching a maximum of 25 °C. Figure 3b shows the temperature change curve of the antibacterial solution under different light power densities of NIR light with a fixed antibacterial solution concentration (0.1 mg/mL). The experimental results showed that the higher the light power density, the more obvious the temperature increase in the antibacterial solution. The effect of power change was more significant than that of concentration change on the temperature increase. To visualize this, an infrared camera was used to capture the temperature change in the antibacterial solution. Figure 3c shows the NIR imaging data of the LM@Van nanoparticle solutions at different time points, indicating that the temperature change was significant within the first minute before light exposure, consistent with the temperature curve in Figure 3a. After 300 s of light exposure, the solution temperature reached 53.3 °C, fully demonstrating the excellent photothermal conversion performance of LM@Van. Meanwhile, LM@Van exhibits good stability. The nanostructure remains unchanged before and after light exposure, and the particle size does not change when stored at room temperature. In addition, the temperature of LM@Van can be controlled by adjusting the laser power density or nanoparticle concentration, which is beneficial for the precise control of the antimicrobial process.

**Figure 4:**
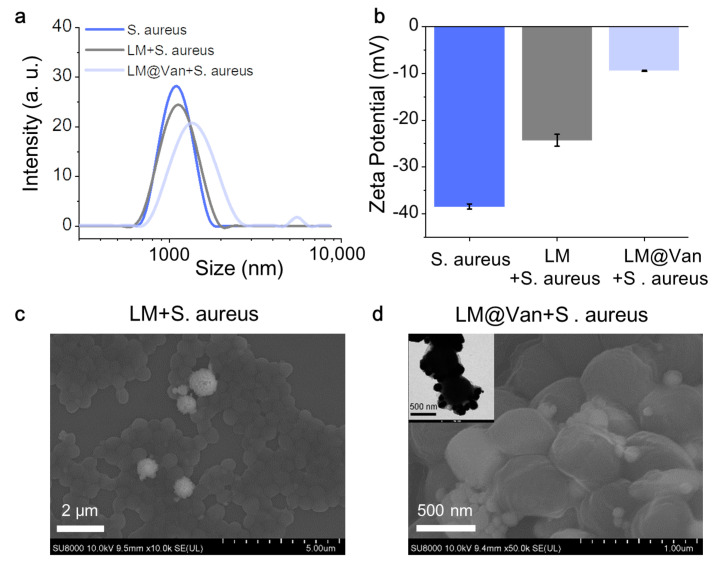
Characterization of interactions between LM@Van nanoparticles and *S. aureus*. (**a**) Particle size distribution of *S. aureus*, LM/*S. aureus*, and LM@Van/*S. aureus*; (**b**) zeta potential of *S. aureus*, LM/*S. aureus*, and LM@Van/*S. aureus*; (**c**) microscopic characterization of LM/*S. aureus* particles; (**d**) microscopic characterization of LM@Van/*S. aureus* particles.

Furthermore, to demonstrate the targeting ability of the antibacterial nanomaterial system, it is necessary to characterize the interaction between the antibacterial material and bacteria. Figure 4a shows the particle size distribution of *S. aureus* and different antibacterial materials, indicating that the average particle size of *S. aureus* was 1 μm, and the particle size of the LM nanoparticle and bacterial mixture did not change significantly. However, the LM nanoparticle modified with Van increased in size to 1.5 μm after being added to bacteria due to the targeting effect of Van, suggesting that the nanoparticles were enriched on the bacterial surface. Figure 4b shows the change in the zeta potential of different solution systems. Due to the surface’s negatively charged acidic phospholipid components, the bacteria’s potential was—38 mV. After the addition of LM@Van, the potential moved toward the positive direction due to the large number of amino groups on the surface of Van, reaching approximately—9 mV. This shift in zeta potential is a clear indication that *S. aureus* can bind well to vancomycin-coated liquid metal. Such a shift in zeta potential is a strong indication that *S. aureus* can bind well to Van-coated LM, further proving the targeted antibacterial ability of this nanomaterial system.

Additionally, we characterized the apparent morphology when bacteria and different antibacterial materials interacted. As shown in Figure 4c, the bacteria were spread on the substrate surface, and LM nanoparticles were randomly distributed in different positions, indicating that there was no significant interaction between the bacteria and LM. Experimental results revealed that antimicrobial agents consisting of LM only did not demonstrate a targeting effect on bacteria. However, with the addition of Van, a significant accumulation of bacteria was found. As illustrated in Figure 4d, a large amount of LM@Van particles were observed at the bacterial connection site, indicating that LM@Van had the function of inducing bacterial aggregation. The upper left corner of Figure 4d is the TEM image of the interaction between bacteria and LM@Van, which shows that LM@Van was enriched on the membrane surface at the single bacterial layer level, further evidencing that Van imparts targeting properties to LM-based antimicrobial agents.

**Figure 5:**
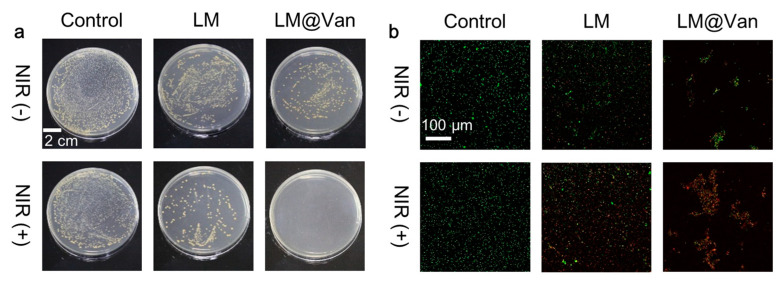
Antibacterial properties of LM@Van nanoparticles. (**a**) Antibacterial effect of LM and LM@Van with and without NIR; (**b**) results of bacterial live/dead staining after LM and LM@Van treatment, where green represents live bacteria and red represents dead bacteria. No substances were added to the control group.

As illustrated in Figure 5, the antibacterial properties of the nanomaterial system were assessed. Figure 5a depicts a monoculture bacterial plate treated with different materials. The antibacterial effect of LM or LM@Van is not significant without NIR laser irradiation. In the absence of 808 nm NIR light irradiation, the LM nanoparticle solution exhibits a certain degree of antibacterial effect due to the accelerated release of Ga ions from the nanoparticles. As a comparison, the LM nanoparticle solution modified with Van exhibits improved antibacterial properties. Under the stimulation of 808 nm NIR light, both LM and LM@Van nanoparticle solutions exhibit reduced bacterial counts, indicating a significant enhancement in the antibacterial ability of the material system. Specifically, LM@Van shows the most significant effect, with almost complete bacterial killing compared to the control experiment, which indicates that the antibacterial performance has nearly reached 100%, demonstrating the effectiveness of the antibacterial strategy. The bacterial live/dead staining results after treatment with different materials are shown in Figure 5b, where the green represents live bacteria and the red indicates dead bacteria. After treatment with LM@Van, there is a significant increase in dead bacteria, and the death rate is further increased after 808 nm NIR stimulation. Meanwhile, the bacterial live/dead staining results also show that LM@Van causes obvious bacterial aggregation, reaffirming LM@Van’s targeted antibacterial properties. These experimental tests are consistent with the previous analytical results, further indicating that the LM@Van antimicrobial material system has certain targeting and photothermal enhancement characteristics. In the future, more antimicrobial materials with a core–shell structure similar to LM@Van could be prepared along this direction. To achieve targeting, the antibiotics should be considered as the shell of nanoparticles.

## 4. Conclusions

In summary, we have successfully developed a targeted nanomaterial antibacterial agent that combines Van with LM, and its antibacterial effect has been further enhanced via photothermal mechanisms. Firstly, we demonstrated that Van can be effectively coated onto the surface of LM using ultrasound, as confirmed via microscopic observations and zeta potential testing. Subsequently, we elucidated the targeted effect of this antibacterial material by revealing the interface interactions between the selected bacteria and this nanomaterial. Also, the excellent photothermal conversion efficiency of LM was demonstrated through a temperature rise curve and infrared testing. Antibacterial experiments showcased the superior antibacterial performance of Van-coated LM nanoparticles, while the application of near-infrared light significantly enhanced the antibacterial effect of this material system, even achieving 100% antibacterial efficacy. Importantly, the Van used in this study can be replaced with other narrow-spectrum antibacterial agents, thus opening up more new possibilities for the development of LM-based antibacterial agents with core–shell structures and advancing this research direction further. It is expected that the study will provide fundamental data and potential applications for subsequent clinical practice.

## Data Availability

Data are contained within the article.

## Supplementary Materials

The following supporting information can be downloaded at https://www.mdpi.com/article/10.3390/bioengineering10070748/s1: Figure S1: Characterization and toxicity assays of LM@Van.; Figure S2: the original TEM image of LM@Van.
