# Supplementary material for: A Photothermally Enhanced Vancomycin-Coated Liquid Metal Antimicrobial Agent with Targeting Capability
Source: Bioengineering (Basel). 2023 Jun 22;10(7):748. doi: 10.3390/bioengineering10070748 (PMC10376194; doi:10.3390/bioengineering10070748)
Supplement: Supplementary file 1 [file bioengineering-10-00748-s001.zip › bioengineering-2418194-supplementary.pdf]

A photothermally enhanced Vancomycin-coated liquid metal antimicrobial agent with targeting capability

Bo Wang [1], Sen Chen [2, *], Xuyang Sun [1], Xiaohui Shan [2], Xiyu Zhu [2], Bo Yuan [2], Hongzhang Wang [4], Gang Zhou [1, *] and Jing Liu [2, 3, *]

[1] School of Biological Science and Medical Engineering, Beihang University, Beijing 100083, China
[2] Department of Biomedical Engineering, School of Medicine, Tsinghua University, Beijing 100084, China

[3] Beijing Key Lab of Cryo-Biomedical Engineering, Technical Institute of Physics and Chemistry Chinese Academy of Sciences, Beijing 100190, China
[4] Center of Double Helix, Tsinghua Shenzhen International Graduate School, Tsinghua University, Shenzhen 518055, China

*Correspondence: chensen@tsinghua.edu.cn (S.C.); zhougang@buaa.edu.cn (G.Z.); jliubme@tsinghua.edu.cn (J. L.)

Keywords: liquid metal; vancomycin; targeted antibacterial; photothermal conversion

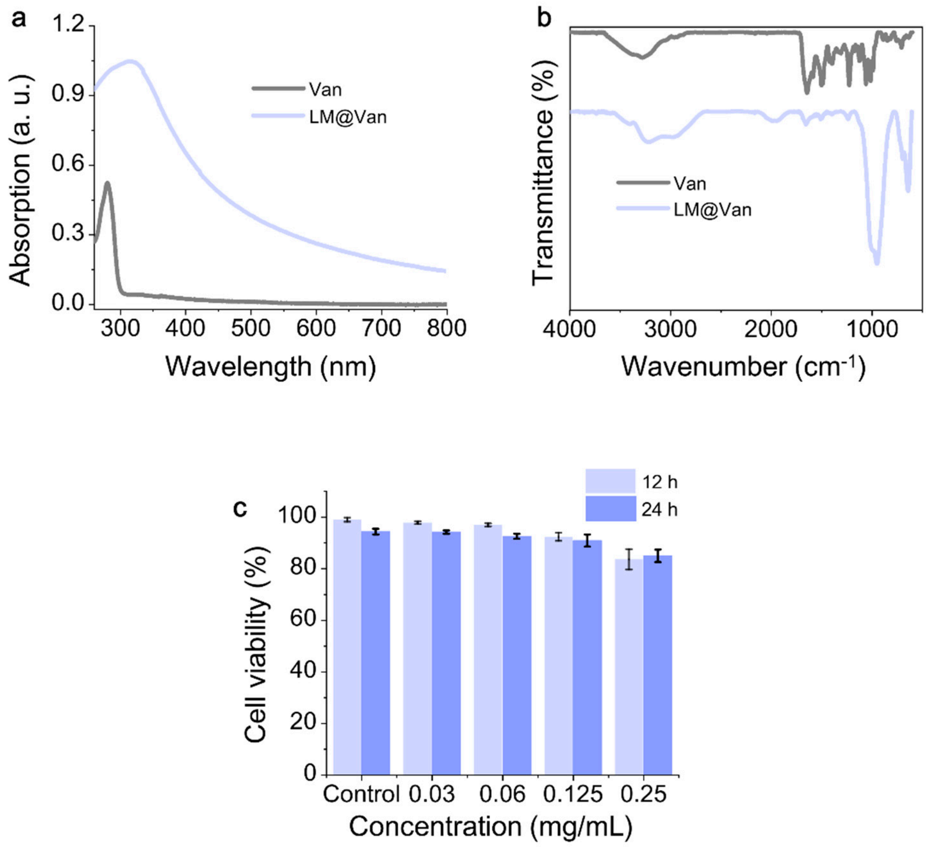

Figure S1. Characterization and toxicity assays of LM@Van. (a) UV-vis spectra of Van and LM@Van. (b) FTIR spectra of Van and LM@Van. (c) Cellular activity experiments with different concentrations of LM@Van.

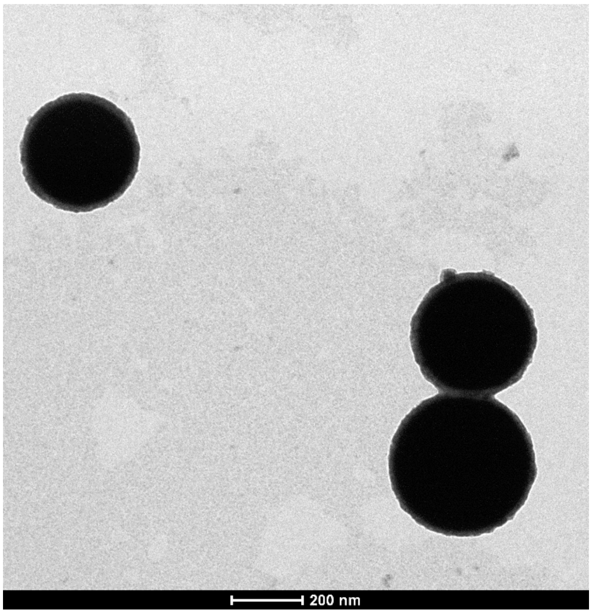

Figure S2. The original TEM image of LM@Van.
